# Selenium hyperaccumulators harbor a diverse endophytic bacterial community characterized by high selenium resistance and plant growth promoting properties

**DOI:** 10.3389/fpls.2015.00113

**Published:** 2015-03-02

**Authors:** Martina Sura-de Jong, Ray J. B. Reynolds, Klara Richterova, Lucie Musilova, Lucian C. Staicu, Iva Chocholata, Jennifer J. Cappa, Safiyh Taghavi, Daniel van der Lelie, Tomas Frantik, Iva Dolinova, Michal Strejcek, Alyssa T. Cochran, Petra Lovecka, Elizabeth A. H. Pilon-Smits

**Affiliations:** ^1^Department of Biochemistry and Microbiology, University of Chemistry and Technology in PraguePrague, Czech Republic; ^2^Life Sciences and Technology, Van Hall Larenstein University of Applied SciencesLeeuwarden, Netherlands; ^3^Biology Department, Colorado State UniversityFort Collins, CO, USA; ^4^FMC Corporation, Center of Excellence for Agricultural Biosolutions, Research Triangle ParkNC, USA; ^5^Institute of Botany, Academy of Sciences of the Czech RepublicPruhonice, Czech Republic; ^6^The Institute for Nanomaterials, Advanced Technology and Innovation, Technical University of LiberecLiberec, Czech Republic

**Keywords:** selenium, hyperaccumulator, endophyte, bacteria, phytoremediation, T-RFLP, microbial diversity

## Abstract

Selenium (Se)-rich plants may be used to provide dietary Se to humans and livestock, and also to clean up Se-polluted soils or waters. This study focused on endophytic bacteria of plants that hyperaccumulate selenium (Se) to 0.5–1% of dry weight. Terminal restriction fragment length polymorphism (T-RFLP) analysis was used to compare the diversity of endophytic bacteria of hyperaccumulators *Stanleya pinnata* (Brassicaceae) and *Astragalus bisulcatus* (Fabaceae) with those from related non-accumulators *Physaria bellii* (Brassicaceae) and *Medicago sativa* (Fabaceae) collected on the same, seleniferous site. Hyperaccumulators and non-accumulators showed equal T-RF diversity. Parsimony analysis showed that T-RFs from individuals of the same species were more similar to each other than to those from other species, regardless of plant Se content or spatial proximity. Cultivable endophytes from hyperaccumulators *S. pinnata* and *A. bisulcatus* were further identified and characterized. The 66 bacterial morphotypes were shown by MS MALDI-TOF Biotyper analysis and 16S rRNA gene sequencing to include strains of *Bacillus, Pseudomonas, Pantoea, Staphylococcus, Paenibacillus, Advenella, Arthrobacter*, and *Variovorax*. Most isolates were highly resistant to selenate and selenite (up to 200 mM) and all could reduce selenite to red elemental Se, reduce nitrite and produce siderophores. Seven isolates were selected for plant inoculation and found to have plant growth promoting properties, both in pure culture and when co-cultivated with crop species *Brassica juncea* (Brassicaceae) or *M. sativa*. There were no effects on plant Se accumulation. We conclude that Se hyperaccumulators harbor an endophytic bacterial community in their natural seleniferous habitat that is equally diverse to that of comparable non-accumulators. The hyperaccumulator endophytes are characterized by high Se resistance, capacity to produce elemental Se and plant growth promoting properties.

## Introduction

Endophytic and rhizospheric microorganisms play an important role in plant physiology, including nutrient acquisition and abiotic and biotic stress resistance (Weyens et al., 2013). Endophytic microbes may colonize the interior of any plant part, including the root, stem, leaves, flowers and seeds (Jha et al., 2013). The highest densities of endophytic microorganisms have been observed in the roots, decreasing from root to stem to leaves (Moore et al., 2006). Many bacterial endophytes are closely related to common soil rhizosphere bacteria such as *Enterobacter, Pseudomonas, Burkholderia, Bacillus, Azospirillum, Serratia, Stenotrophomonas, Methylobacterium, Paenibacillus, Streptomyces, Rhizobium, Rhodococcus, Arthrobacter, Variovorax*, and others (Miller et al., 1998; Lodewyckx et al., 2002; Strobel et al., 2004; Guan et al., 2005; Taghavi et al., 2005; Ryan et al., 2008; Weyens et al., 2013; Croes et al., 2014). The diversity of endophytes is dependent on plant species, cultivar and probably cultivation conditions (Ulrich et al., 2008).

Rhizospheric and endophytic microorganisms form microbial communities important for plant growth and development. These bacteria can affect plant growth by different direct and indirect mechanisms (Gupta et al., 2000; Glick, 2012), including (1) increased mineral nutrient access or bioavailability, including nitrogen fixation; (2) repression of soil-borne pathogens (by the production of hydrogen cyanide, siderophores, antibiotics, and/or competition for nutrients); (3) improving plant stress tolerance to drought, salinity and metal toxicity; and (4) production of phytohormones such as indole-3-acetic acid (Gupta et al., 2000; Jha et al., 2013). In addition, several studies have shown that not only rhizospheric, but also endophytic bacteria have the potential to enhance the removal of soil contaminants by phytoremediation, especially organic contaminants (Germaine et al., 2003; Barac et al., 2004; Compant et al., 2005; Dowling et al., 2008; Doty et al., 2009; Taghavi et al., 2010). The positive effects of endophytes on plant growth and elemental accumulation may be utilized in various applications. Inoculation of plant species with selected endophytes may achieve higher biomass production for agricultural crops, may confer protection of these crops against pathogens or abiotic stresses, and may increase a crop's nutritional value (biofortification) and ability for environmental cleanup (phytoremediation) (Pilon-Smits, 2005).

An interesting group of plants for phytoremediation are the so-called hyperaccumulator species, which accumulate one or more inorganic, toxic elements to levels upwards of 100-fold higher than other species growing under the same conditions (Cappa and Pilon-Smits, 2014). The toxic elements As, Co, Cu, Mn, Ni, Pb, Se, and Zn can be hyperaccumulated to 0.1–4% of dry weight, usually both in root and shoot (Cappa and Pilon-Smits, 2014). Since plants always live in close relation with microbial communities, the question may be raised how hyperaccumulation is affected by plant-associated microbes, and *vice versa*, how hyperaccumulation affects microbial density and composition. Plant-microbe interactions of hyperaccumulators are a relatively unexplored area (Alford et al., 2010). Studies on Ni-hyperaccumulation have shown that rhizosphere microorganisms affected plant gene expression, as evident from differences in shoot proteome (Farinati et al., 2009, 2011). Inoculation of hyperaccumulator *Sedum alfredii* with *Burkholderia cepacia* (Li and Wong, 2012) did not enhance plant growth or metal uptake, but enhanced metal translocation of Cd and Zn as well as metal tolerance. Thus, when bacteria are inoculated to a hyperaccumulator, plant physiology may undergo changes. Conversely, plants affect their endophytic communities. Chen et al. (2014) reported that both plant species and heavy metal pollution contributed to the shaping of the dynamic endophytic bacterial communities associated with hyperaccumulators.

Selenium hyperaccumulators were shown to contain bacterial and fungal endophytes, including Rhizobia in root nodules as well as a seed coat fungus in the legume hyperaccumulator *Astragalus bisulcatus* (Valdez Barillas et al., 2012). These microbes were hypothesized to be responsible for the high levels of elemental Se (up to 30% of tissue Se) observed in nodules, roots, seeds and stems of *A. bisulcatus* in the field (Lindblom et al., 2013). The high Se levels in Se hyperaccumulators may also affect the local microbial ecology in seleniferous areas. High-Se litter (550 mg kg^−1^ DW) was found to decompose faster than low-Se litter, and to harbor higher levels of culturable microbes, suggesting the presence of a community of Se-resistant microbial decomposers (Quinn et al., 2011). On the other hand, Se was found to protect plants from Se-sensitive fungal pathogens (Hanson et al., 2003). Thus, Se hyperaccumulators may negatively affect microbial partners if these are Se sensitive, while at the same time offering an exclusive niche for Se-resistant microbial partners. This trend has also been observed for other ecological partners, including herbivores, plants and pollinators (as reviewed in Quinn et al., 2010; El-Mehdawi and Pilon-Smits, 2012).

The aims of the current study were to determine how the microbiome differs on the endophyte level between between Se hyperaccumulator species and related non-hyperaccumulator species on the same seleniferous site, and to characterize endophytic bacteria from Se hyperaccumulators with respect to Se-related properties and plant growth promoting properties. The significance of these studies is two-fold. They are among the first to characterize the endophytic microbiomes of plants that hyperaccumulate extraordinary levels of toxic elements. In addition, microbes found to have extreme Se resistance or Se metabolic properties, or plant growth promoting properties, may have applications in industry and agriculture. Isolated microbes with high Se tolerance and ability to produce elemental Se may for instance be used for wastewater treatment and/or the production of Se nanoparticles (Staicu et al., 2015). Microbes that boost plant growth and/or plant Se tolerance and accumulation may benefit the practices of Se phytoremediation and Se biofortification, which would have substantial significance, since both Se toxicity and Se deficiency are serious problems worldwide (Zhu et al., 2009; Bãnuelos et al., 2011).

## Materials and methods

### Collection of plant material

Seeds of *Medicago sativa* were obtained from a local nursery. *Brassica juncea* seeds were obtained from a USDA plant introduction station as described earlier (Harris et al., 2014).

**Figure 1:**
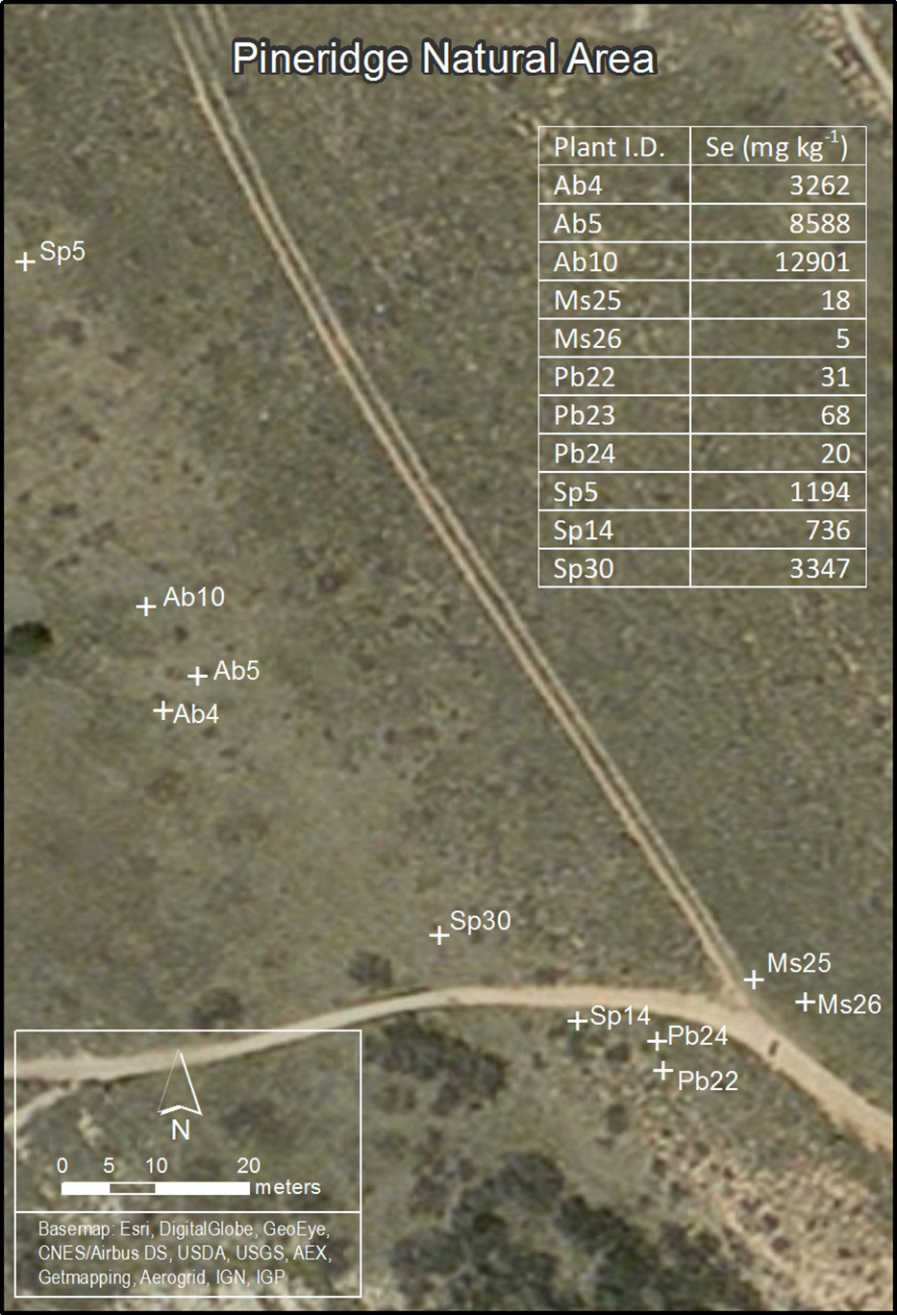
**Map of Pineridge Natural Area, the seleniferous site west of Fort Collins, CO, USA where plants were sampled for T-RFLP analysis**. Ab4, Ab5, Ab10—three individual plants of *Astragalus bisulcatus* (hyperaccumulator); Sp5, Sp14, Sp30—three individual plants of *Stanleya pinnata* (hyperaccumulator); Ms25, Ms26—two individual plants of *Medicago sativa* (non-hyperaccumulator); Pb22, Pb23, Pb24—three individual plants of *Physaria bellii* (non-hyperaccumulator). The table insert lists the leaf Se concentration for every plant sampled.

Hyperaccumulators *Astragalus bisulcatus* (Hook) A. Gray and *Stanleya pinnata* (Pursh) Britton plants (roots, stems, leaves) were collected in the summer (August) at Pineridge Natural Area, a seleniferous site west of Fort Collins, CO, USA (Figure 1). This site has been described before (Galeas et al., 2007, 2008). Collected plants were labeled Ab4, Ab5, Ab10, Sp5, Sp14, and Sp30. As non-hyperaccumalating controls, plants (roots, stems, leaves) of *Medicago sativa* L. (samples Ms25, Ms26) and *Physaria bellii* G. Mulligan (samples Pb22, Pb23, Pb24) were collected at the same site. Part of the leaf material was used for total Se concentration measurement (as described below); the rest of the plant material was used for metagenomic studies and isolation of endophytic bacteria. This plant material was stored overnight at 4°C in sterile 10 mM MgSO_4_.

### Measurement of total Se concentrations

The concentration of total Se was measured using inductively coupled plasma atomic emission spectrometry (ICP-AES) according to Fassel (1978). The leaves of collected plants were rinsed with distilled water and dried for 48 h at 55°C. Hundred micro gram of samples were digested in nitric acid as described by Zarcinas et al. (1987).

### Sterilization of the plant surface

Plant material was surface-sterilized for 15 min slightly shaken in 2% active hypochlorite (household bleach, 4× dilution) containing 0.5 ml L^−1^ Tween 20. Three wash steps with sterile distilled water were performed. A sample of the water from the last wash step was streaked on solid Luria Bertani (LB) media to verify sterility.

### Isolation of metagenomic DNA and T-RFLP

For endophyte comparative community diversity analysis, surface-sterilized plant material was homogenized under sterile conditions using liquid nitrogen, mortar and pestle. For each plant, equal weight samples of disintegrated roots, stems and leaves were combined into one tube and deep frozen. The metagenomic DNA (DNA of the plant body, containing a plant nuclear, plastid and mitochondrial DNA, as well as endophytic bacterial and fungal DNA) was isolated with the DNeasy Plant Mini Kit (Qiagen, USA). Then, part of the gene encoding for 16S rRNA was amplified with primers 26F-FAM (5′ labeled with 6-karboxyfluorescein) and 1114R (26F-FAM 5′- AGA GTT TGA TCM TGG CTC A-3′, 1114R 5′- GGG TYK CGC TCG TTR-3′) following a program of 95°C for 2 min, then 35 cycles of 95°C for 20 s, 63°C for 30 s, and 70°C for 30 s, and final extension at 70°C for 7 min. The 50-μL reaction mixtures contained template DNA, 5 pmol of each primer (Generi Biotech, Czech Republic), 5 nmol deoxynucleotide triphosphates (dNTPs), 2.5 μg bovine serum albumin (BSA), and 0.5 U Novagen® KOD Hot Start polymerase with the corresponding buffer (Merck, Germany). Further, a reconditioning step was performed. Here 5-μL aliquots of the initial PCR product were transferred to new reaction mixtures and amplified for three cycles under the same PCR conditions. PCR products were purified with the PureLink® PCR Purification Kit (Invitrogen, USA). Fifty nano gram of DNA were digested with *Hha*I restriction endonuclease following a reaction mixture of template DNA, 4 U *Hha*I (New England BioLabs, UK), 0.3 μL BSA (New England BioLabs, UK) and 2 μL corresponding buffer. The reaction mixture was incubated 60 min at 37°C. After DNA cleavage, 1.5 μL of sodium acetate together with 1 μL of glycogen (molecular biology grade) and 47 μL of 98% ethanol were added. The mixture was incubated 20 min at −80°C to precipitate the DNA. The mixture was centrifuged for 10 min at 4°C at maximum speed. The DNA pellet was washed using first 1 mL of cooled 70% ethanol and a second wash was performed with 98% ethanol.

Analysis of terminal restriction fragment length polymorphism (T-RFLP) was performed by fragmentation analysis. Aliquots of the restricted fragments were mixed with an internal size standard (LIZ600, Applied Biosystems) and separated on an automated genetic analyzer using fragmentation module (Applied Biosystems 3500 Genetic Analyzer). The data were processed in GeneMapper 5.0 software (Applied Biosystems, Foster City, CA) in which peak detection was done by the default settings (Local Southern method with Peak Amplitude Threshold of 50 fluorescence units, no smoothing and Baseline Window of 51 points). Peak tables from all spectra were exported to MS Excel, where the peak positions were rounded to the closest integer and samples were normalized by dividing each peak's fluorescence intensity by total signal intensity of the corresponding sample. All terminal restriction fragments (T-RFs) with size lower 50 bp and intensities below 0.01 were excluded to evaluate the differences in bacterial diversity of hyperaccumulators and non-hyperaccumulator plants.

### Parsimony analysis to compare relatedness of endophyte microbiome samples

Equally weighted parsimony tree searches were conducted using Paup^*^ (ver. 4.0b10; Swofford, 2001). Up to 10 trees were held within each of the 2000 random addition tree-bisection-reconnection (TBR) branch swapping searches. Branch support was determined using parsimony jackknife (Farris et al., 1996); analyses were conducted with the removal probability set to approximately e^−1^ (0.37).

### Isolation of endophytic bacteria

Disintegration of surface-sterile plant material (separate roots, stems and leaves) was carried out at room temperature in 1.5 mL microcentrifuge tubes using a plastic micropestle. Samples were homogenized in sterile 10 mM MgSO_4_ (dilution 10^−1^). Hundred micro liter of dilutions of 10^−1^, 10^−3^ and 10^−5^ in 10 mM MgSO_4_ were spread out on Petri dishes with solid half-strength LB medium (½LB), ½LB with plant extract (preparation of plant extract described below), and on ½MS plant cultivation media (Murashige and Skoog, 1962). Bacteria were cultivated at room temperature for 7 days. For each plant species, microorganisms displaying different morphologies were re-streaked on new plates to obtain axenic monocultures.

### Preparation of plant extract

Plant extracts of *A. bisulcatus* and *S. pinnata* were prepared from 2.8 g of fresh leaf material by disintegration with mortar and pestle and adding 40 mL of warm water (~40°C). This mixture was centrifuged for 5 min at 4000 × g and the supernatant was filter sterilized (0.22 μm pore size). The obtained volume of each plant extract was 16 mL. The ratio of this plant material used in the cultivation medium represented 1:50 (v/v).

### Morphological analysis of isolates

Isolates were characterized according to their macroscopic and microscopic morphology (form/elevation/margin/surface/color of the colony, bacilli/cocci/gram staining). Detrended canonical correspondence analysis (DCA) was applied to explore the correlation between morphological characteristics of endophytic isolates using CANOCO 5 according to ter Braak and Šmilauer (1998). Plant organs (root, stems, and leaves) from which the isolates were isolated were used as supplementary explanatory variables.

### Identification of endophytic bacteria using MALDI-TOF MS

Isolates were identified via the method of matrix-assisted laser desorption ionization - time of flight (MALDI-TOF) mass spectrometry and MALDI Biotyper. The Bruker Biflex IV MALDI-TOF spectrometer (equipped with a UV nitrogen laser [337 nm] and a dual microchannel microplate detector) and MALDI Biotyper 2.0 software (Bruker Daltonics, Bremen, Germany) were used. Samples for the analysis were prepared according to manufacturers' recommendations: after 24–48 h of cultivation of an isolate on LB medium (Oxoid Ltd., United Kingdom) at 28°C, a single colony was transferred with a sterile tip onto the MALDI target in triplicates, drizzled with 1 μL of a saturated solution of α-cyano-4-hydroxycinnamic acid (Sigma-Aldrich) in organic solution (50% acetonitrile, 2.5% trifluoroacetic acid), and directly screened. The measurement of the spectra was performed as previously described (Uhlik et al., 2011). The matching of unknown spectra to the reference database is based on dedicated score (point) values. This value is used for calculating the final score, according to which the identification results are evaluated as follows: if the logarithmic value of the final score is between 2.3 and 3, the isolate is identified at the level of species; for values between 2 and 2.3, the identification is secure at the level of genus; for values between 1.7 and 2, the identification at the level of genus is probable; and for values lower than 1.7, the identification is not successful.

### Identification of endophytic bacteria using 16S rRNA gene sequencing

Several bacterial isolates were identified using 16S rRNA gene sequence analysis. A colony PCR was performed to amplify part of the gene encoding 16S rRNA with primers 8F (5′- AGA GTT TGA TCC TGG CTC AG- 3′, Lane, 1991) and 926R (5′- CCG TCA ATT CCT TTR AGT TT- 3′, Amann et al., 1995) following a program of 96°C for 3 min, 10 cycles of 96°C for 45 s, 50°C for 30 s and 72°C for 2 min, 25 cycles of 96°C for 20 s, 50°C for 30 s and 72°C for 2 min. The 25-μL reaction mixtures contained the template DNA, 5 pmol of each primer (Generi Biotech, Czech Republic), 5 nmol deoxynucleotide triphosphates (dNTPs) (New England Biolabs), 2.5 μg bovine serum albumin (BSA) (New England Biolabs), and 0.5 U Taq DNA polymerase with the corresponding buffer (New England Biolabs). PCR products were purified with the QIAquick PCR purification kit (Qiagen, Germany). Purified products were sent to University of Chicago Research Center DNA Sequencing Facility, USA. Classification was performed by means of Ribosomal Database Project (RDP) Classifier (14) at an 80% confidence threshold.

### Functional characterization of endophytic isolates

Selenate (SeO^2−^_4_) and selenite (SeO^2−^_3_) resistance and reduction abilities were tested on solid LB media spiked with 0.1 mM, 1 mM, 10 mM, 100 mM and 200 mM Na_2_SeO_4_ or Na_2_SeO_3_, respectively. The cultivation of bacteria was performed at 28°C overnight, after which growth and color were scored; in absence of good growth, additional monitoring was performed after 3 days. Growth was analyzed qualitatively; the ability to produce red elemental Se was scored visually as red coloration. To analyze the effect of nitrate on selenite reduction and resistance, the bacteria were also grown on 10 mM Na_2_SeO_3_ supplemented with 100 mM KNO_3_, and red color formation, indicative for Se reduction, was scored.

Nitrite reduction ability was tested in liquid media containing 0.1% potassium nitrite (medium composition: beef (meat) extract 3.0 g L^−1^, gelatin peptone 5.0 g L^−1^, potassium nitrite (KNO_2_) 1.0 g L^−1^). The cultivation of bacteria was performed at 28°C (130 rpm). Every 24 h for 6 days in row, 1 mL of the culture was sampled, centrifuged and an aliquot was tested for nitrite presence according to the Griess reaction (Green et al., 1982). If nitrite was present, the reaction mixture turned pink, purple or red (depending on the amount of nitrite present). Reduction of potassium nitrite was shown by a transparent color of the mixture.

Siderophore production of isolates was tested on Chromazurol-S (CAS) agar media as described by Shin et al. (2001). Isolates were cultivated for 120 h at 28°C. Siderophore-producing strains formed a halo zone around the colony. Phosphate solubilization, acetoin production, acid production (methyl red), indole acetic acid (IAA) production, and chitinate and protease activity were analyzed as described by Weyens et al. (2013).

### Plant inoculation with selected bacterial endophytes

*Medicago sativa* and *Brassica juncea* (L.) plants were grown from surface-sterilized seeds on soil collected from Pineridge Natural Area (described by Galeas et al., 2007). After collection, the soil was homogenized and mixed with Turface® in a 2:1 soil: Turface® ratio. Polypropylene (Magenta) boxes were then filled to a height of 2 cm with the mixture. The Magenta boxes were closed and autoclaved for 40 min. Seeds were surface-sterilized by rinsing for 30 min in 15% household bleach (1.5% NaClO) followed by 5 rinses for 5 min each in sterile water, and sown in the Magenta boxes at a density of 3 seeds per box. One week after germination, seedlings were thinned to one plant per box and inoculated with endophytic bacteria as follows. For *M. sativa*, three isolates were used that originated from *A. bisulcatus* (#8, 31, and 32, see **Table 2** for more information). In addition to the single-strain inoculants, a fourth treatment consisted of a mixture of all three isolates, and a fifth control treatment received no inoculum. For *B. juncea*, four isolates were used that originated from *S. pinnata* (#54, 64, 71, and 77, see **Table 2** for more information). These were inoculated individually as well as in a mix of all four, and there was an uninoculated control treatment. The bacteria were grown in half-strength LB for 24 h at 25°C, harvested by centrifugation and resuspended in 10 mM MgSO_4_ to an OD_600_ of 1.0. One mL of inoculum was delivered using a pipette to the base of the seedlings; the controls received 1 mL of 10 mM MgSO_4_. The plants were allowed to grow for 6 weeks. The boxes were watered with autoclaved water every 2 weeks (twice total). The plants were then harvested, separating the root and shoot. Small shoot and root samples from each plant were placed in 10 mM MgSO_4_ for re-isolation of bacterial endophytes, to verify successful inoculation. These were ground using sterile micropestles in microcentrifuge tubes, and 100 μL of the extract was streaked onto LB agar plates, which were monitored after 24 h and compared visually with the inoculum. The remainder of the root and shoot material was dried and weighed. Root and shoot samples were digested in nitric acid (Zarcinas et al., 1987) and analyzed for Se concentration using inductively coupled plasma optical emission spectrometry (ICP-OES) according to Fassel (1978) and as described by Harris et al. (2014).

## Results

### Analysis of bacterial endophytes using a metagenomic approach

Plant leaves, stems and roots of three Se hyperaccumulator (HA) species *Astragalus bisulcatus* (Fabaceae) and *Stanleya pinnata* (Brassicaceae) and related non-hyperaccumulator (non-HA) species from the same site, *Medicago sativa* (Fabaceae) and *Physaria bellii* (Brassicaceae) were collected in triplicate and examined for microbial endophytes. To determine if the diversity of bacterial endophytes can be correlated to the Se content or geographic location of the collected plants, Se concentration in leaves was determined, and the locations of the sampled plants was mapped (Figure 1). As expected, the HA species *S. pinnata* and *A. bisulcatus* contained much higher Se levels (averaging around 1700 and 8000 mg kg^−1^ DW, respectively) than the non-HA species (averaging around 20 and 40 mg kg^−1^ DW).

**Figure 2:**
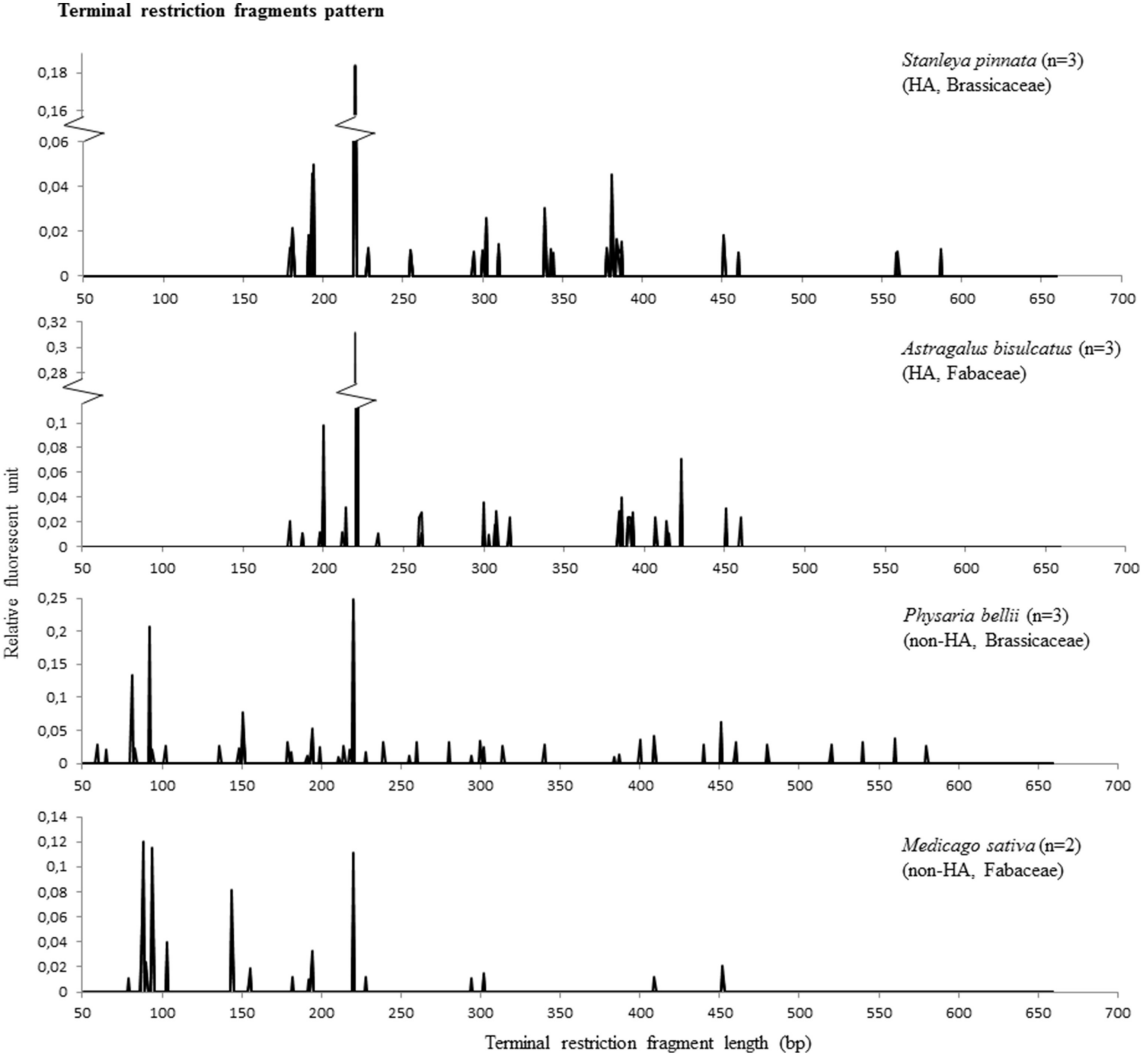
**T-RFLP results representing microbial diversity in endophytes of hyperaccumulator species *S. pinnata* (Brassicaceae) and *A. bisulcatus* (Fabaceae) as well as non-hyperaccumulators collected from the same seleniferous site, *P. bellii* (Brassicaceae) and *M. sativa* (Fabaceae)**. Each RFLP pattern shows the collective peaks from 3 plants, except for *M. sativa*, where the pattern was comprised from two plants (the third did not give any peaks).

To examine the diversity of the endophytic bacteria of each individual plant, T-RFLP analysis was performed on pooled root, stem and leaf DNA. The T-RF pattern may serve as a proxy for microbial diversity, although the T-RFs can contain one or more bacterial species. The used primers (26F-FAM and 1114R) amplified also mitochondrial DNA, which resulted in a T-RF of 220-221 bp after the restriction digest. The number of T-RFs (Supplemental Table 1) obtained per individual plant was not significantly different between the four plant species (ANOVA, *p* > 0.05), and was 12.5, 16.7, 17.3, and 18.7 for *M. sativa, S. pinnata, P. bellii*, and *A. bisulcatus*, respectively. Some apparent differences between HA and non-HA species are that both non-HA species showed an abundance of peaks of <100 bp length, while those peaks were not present in HA (Figure 2). Both HA species showed an apparent enrichment of terminal restriction fragments of sizes ~200 bp, ~380 bp and in the case of *A. bisulcatus* also 423 bp (Figure 2). Additionally, the T-RF profiles from members of the Brassicaceae (HA and non-HA) contained terminal fragments of 559 bp and 587 bp, whereas these fragments were not present in the profiles of the two members of the Fabaceae family.

**Figure 3:**
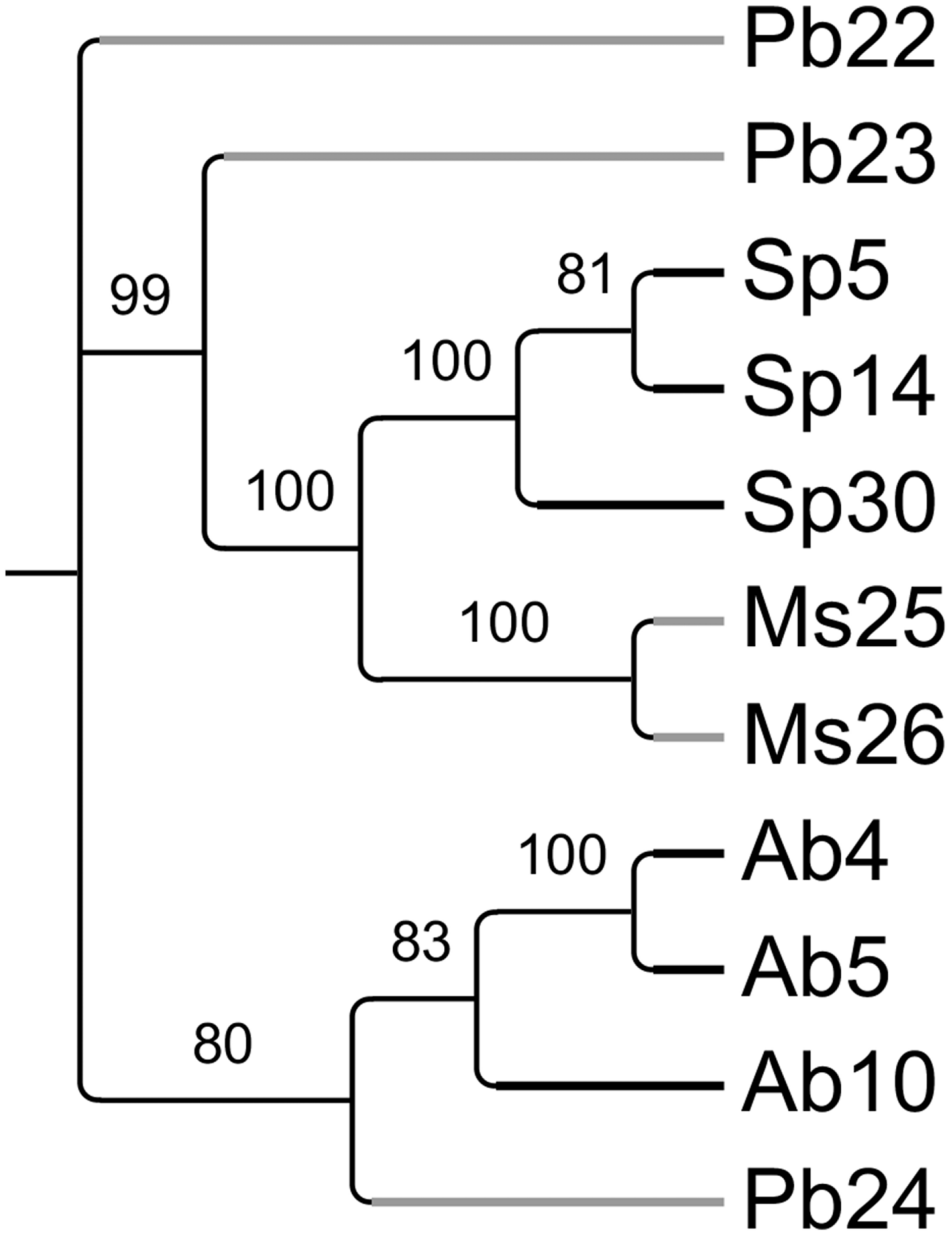
**Most parsimonious tree reflecting similarity in endophyte diversity between the sampled plants (PAUP, obtained from T-RFLP data matrix listed in Supplemental Table 1)**. Values above the branches represent parsimony jackknife support values ≥50%. *Physaria bellii* 22 was used to root the tree. Black branches represent hyperaccumulators and gray branches represent non-hyperaccumulators.

To compare the microbiomes of the various plant samples in more detail, a matrix (Supplemental Table 1) scoring the presence or absence of each T-RF in each individual plant was used to create a parsimony tree using PAUP software. A total of 341 characters were analyzed. Seven characters were constant, 64 variable characters were parsimony uninformative and 270 were parsimony informative. As shown in Figure 3, microbiomes obtained from individuals belonging to a certain plant species tended to be similar to each other. This was true even when these individuals were growing at physically remote locations (Figure 1). Beyond the plant species level, there was no apparent similarity between T-RFs patterns, nor did T-RFs patterns cluster according to plant Se content (Figures 1, 3).

### Isolation of bacterial endophytes and their identification

To further investigate the endophytic microbiomes of Se HA species, we cultured endophytic bacteria from *A. bisulcatus* and *S. pinnata*. Three individuals from each species were sampled, and Se levels examined from roots, stems and leaves. For *A. bisulcatus* plant 1 the Se levels were: 1606, 4752, and 8834 mg kg^−1^ DW, respectively. For plant 2 the root, stem and leaf Se levels were 813, 9158, and 13,685 mg kg^−1^ DW, and for plant 3 they were 694, 5658, and 4732 mg kg^−1^ DW, respectively. The root, stem and leaf Se levels for *S. pinnata* plant 1 were 417, 1655, and 277 mg kg^−1^ DW, respectively; for plant 2 they were 847, 3384, and 3713 mg kg^−1^ DW, respectively, and for plant 3 they were 944, 583, and 2406 mg kg^−1^ DW, respectively.

*Astragalus bisulcatus* and *S. pinnata* endophytic bacteria were isolated separately from stems, leaves and roots. Most bacteria were isolated from roots, followed by leaves, then stems. In total, 54 and 50 isolates were obtained from *A. bisulcatus* and *S. pinnata*, respectively. *A. bisulcatus* stems, leaves and roots yielded 4, 18, and 32 isolates, while 8, 8, and 34 isolates were obtained from *S. pinnata* stems, leaves and roots, respectively.

**Figure 4:**
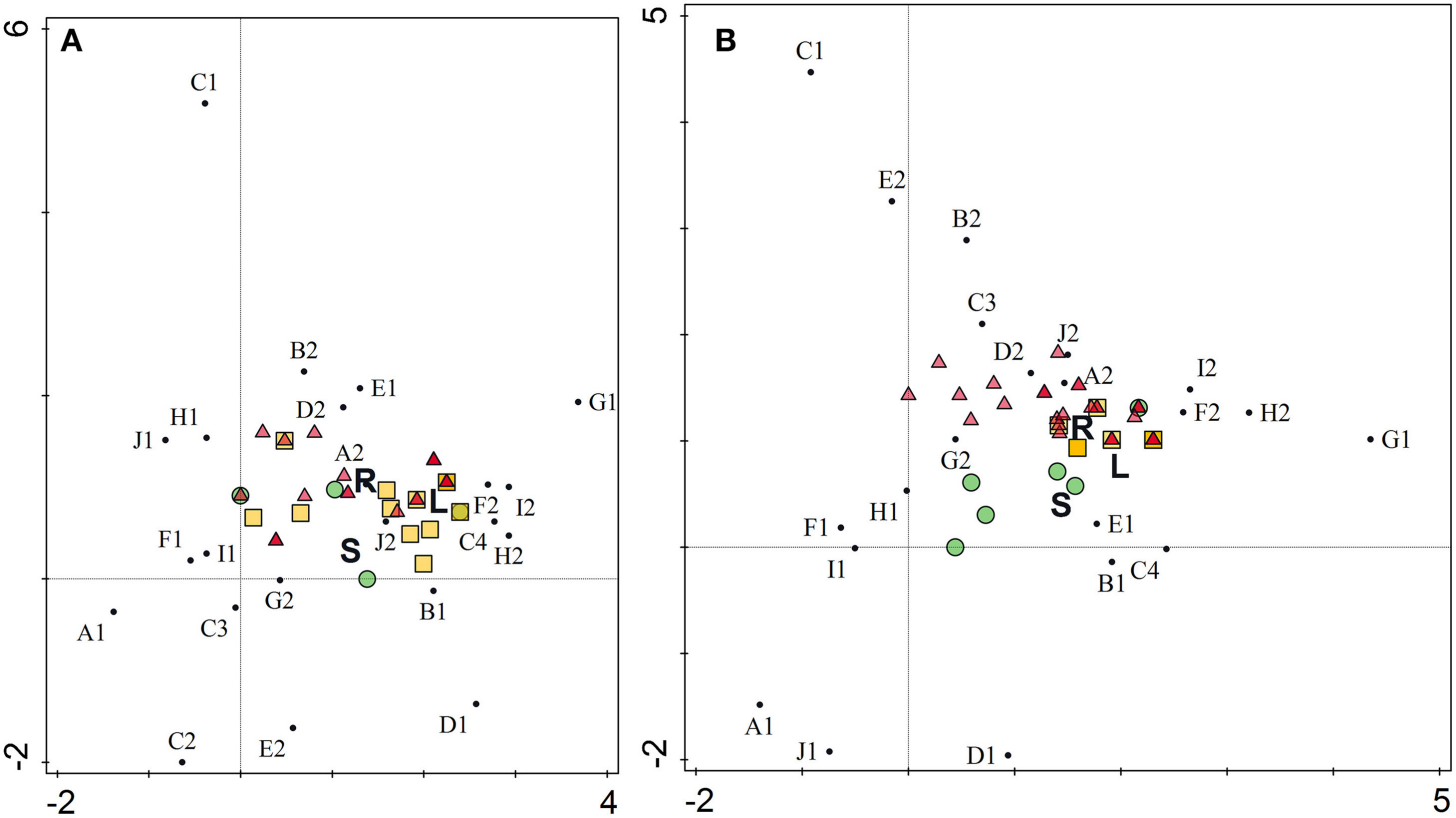
**Ordination diagram as an output of detrended canonical correspondence analysis of morphological characteristics of endophytic isolates from hyperaccumulators *Astragalus bisulcatus* (A) and *Stanleya pinnata* (B)**. X axis, DCA axis1; Y axis, DCA axis2. Organ variables are represented by letters (R, root; S, stem; L, leaf), isolates by colored circles (isolates from stems), triangles (isolates from roots) and squares (isolates from leaves) and characteristics by small black circles. A1 (cocci), A2 (rods), B1 (Gram-positive), B2 (Gram-negative), C1 (yellow), C2 (yellowish), C3 (creamy), C4 (white), D1 (colonies getting brown with time), D2 (colonies do not get brown with time), E1 (circular colony form), E2 (irregular form), F1 (entire margin), F2 (undulate margin), G1 (flat colony), G2 (raised colony), H1 (smooth), H2 (rough), I1 (shiny), I2 (dry), J1 (punctiform), J2 (regular size).

Macroscopic and microscopic characterization of individual isolates was performed (Supplemental Table 2) and detrended canonical correspondence analysis (CANOCO) used to explore any correlation between endophytic isolates composition and the plant part from which they were isolated. The ordination diagrams (Figure 4) can be interpreted by the following rule: spatial proximity in the graph reflects similarity. In this manner, similarity and/or correlation among isolates and characteristics can be estimated. Two ordination axes explained 44 and 48% of total variation, while plant organs accounted for only 5 and 6% of variation, respectively. As shown in Figure 4, the macroscopic and microscopic characteristics were not dependent on plant organ for *A. bisulcatus*. In the case of *S. pinnata*, however, isolates from stems had different characteristics than isolates from leaves and roots.

The endophytic isolates were identified via the method of matrix-assisted laser desorption ionization time of flight (MALDI—TOF) mass spectrometry and MALDI Biotyper. Fifty four percent of isolates from *A. bisulcatus* were identified with highly probable species identification, 28% with secure genus identification, probable species identification, 15% with probable genus identification and 3% were not identified. In the case of *S. pinnata*, 42% of isolates were identified with highly probable species identification, 28% with secure genus identification, probable species identification, 26% with probable genus identification and 4% were not identified. Five isolates were also identified using the analysis of 16S rRNA gene sequence. As shown in Table 1, *Bacillus* was the bacterial genus most frequently isolated from the HA plants. Other abundant identified bacterial genera were *Pantoea, Pseudomonas* and *Staphylococcus*; in additional there were isolates of *Paenibacillus, Advenella, Arthrobacter*, and *Variovorax*.

**Table 1:** **Bacterial isolates from hyperaccumulators *Astragalus bisulcatus* and *Stanleya pinnata***.

**Stems**			**Leaves**			**Roots**		
**Isolate**	**Score**	**#**	**Isolate**	**Score**	**#**	**Isolate**	**Score**	**#**
***ASTRAGALUS BISULCATUS***								
*Bacillus atrophaeus*	+++	2	*Bacillus atrophaeus*	+++	7	*Bacillus atrophaeus*	+++	7
*Paenibacillus illinoisensis*	++	1	*Bacillus atrophaeus*	++	4	*Bacillus atrophaeus*	++	2
*Pseudomonas* sp.	+	1	*Bacillus cereus*	++	1	*Bacillus* sp.	+	1
			*Bacillus* sp.	+	3	*Pantoea agglomerans*	+++	10
			*Bacillus sp*.	+/16S^**^	1	*Pantoea agglomerans*	+++/16S^**^	1
			*Pantoea agglomerans*	+++	1	*Pantoea agglomerans*	++	4
			*Staphylococcus epidermidis*	++	1	*Pseudomonas koreensis*	++	1
						*Pseudomonas* sp.	+	2
						*Advenella kashmirensis*	16S^**^	1
						*Variovorax* sp.	+	1
						NR^*^	−	2
***STANLEYA PINNATA***								
*Bacillus atrophaeus*	+++	4	*Bacillus atrophaeus*	+++	5	*Bacillus atrophaeus*	+++	5
*Staphylococcus condimenti*	++	1	*Bacillus atrophaeus*	++	2	*Bacillus atrophaeus*	++	5
*Staphylococcus* sp.	+	1	*Pantoea agglomerans*	+++	1	*Bacillus* sp.	+	6
*Pseudomonas koreensis*	++	1				*Pantoea agglomerans*	+++	6
*Pseudomonas* sp.	+	1				*Pantoea agglomerans*	++	1
						*Pantoea* sp.	+	1
						*Pseudomonas koreensis*	++	3
						*Pseudomonas* sp.	+	2
						*Pseudomonas moraviensis*	16S^**^	1
						*Arthrobacter* sp.	16S^**^	1
						*Staphylococcus* sp.	+	2
						NR^*^	−	2

*Score +++ highly probable species identification, ++ secure genus identification, probable species identification, + probable genus identification, - not reliable identification*.

* NR, not reliable identification;

** *16S, identification by analysis of 16S rRNA gene sequence, #, number of total isolates*.

### Functional characterization of isolated bacterial endophytes

**Figure 5:**
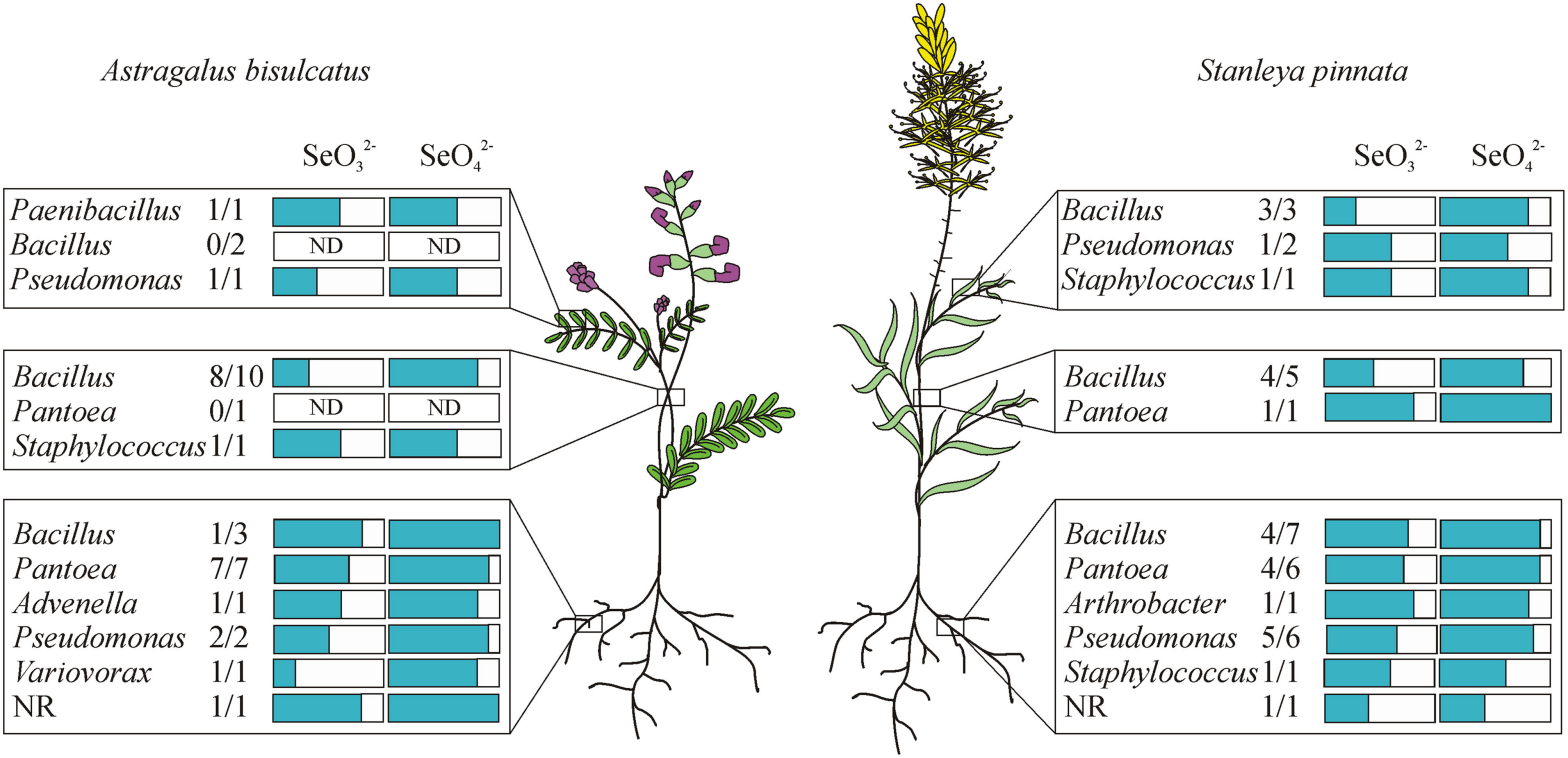
**Distribution of endophytic bacterial isolates in the hyperaccumulators *Astragalus bisulcatus* and *Stanleya pinnata* and their resistance to selenite and selenate (scored from 1 to 5)**. Plants were collected at the Pineridge Natural Area, a seleniferous site west of Fort Collins, CO, USA.

All isolated, purified cultivable strains that were morphologically different (judged from DCA and from the identification using MALDI-TOF MS) were qualitatively screened for their abilities to grow on and/or reduce selenite and selenate, to reduce selenite in the presence of nitrate, to reduce nitrite and to produce siderophores (Table 2). Isolates were grown on LB media containing 0–200 mM SeO^2−^_3_ or SeO^2−^_4_. All isolates were able to grow on both selenite and selenate and reduced selenite to red elemental Se. None of the isolates reduced selenate to red elemental Se. The selenite resistance was scored as follows: 100% of all *A. bisulcatus* and of *S. pinnata* isolates grew on 0.1 mM and 1 mM SeO^2−^_3_, 96% of the *A. bisulcatus* and of *S. pinnata* isolates grew on 10 mM SeO^2−^_3_, 75 and 76% grew on 100 mM SeO^2−^_3_, and 58 and 80% on 200 mM SeO^2−^_3_, respectively. On the two highest concentrations some isolates grew more slowly, needing 3 days instead of one to fully grow. The resistance to selenate was as follows: 96% of *A. bisulcatus* and 88% of *S. pinnata* isolates grew on 0.1 mM SeO^2−^_4_, while 100 and 96% grew on 1 mM SeO^2−^_4_, 100 and 96% on 10 mM SeO^2−^_4_, 100 and 100% on 100 mM SeO^2−^_4_, and 100 and 96% on 200 mM SeO^2−^_4_, respectively. Thus, the resistance of the isolates was generally higher to selenate than to selenite. The same trend was apparent when the selenite and selenate resistance of endophytes isolated from different hosts and organs were given a score (1–5) and the scores were averaged by genus (Table 2, Figure 5). Selenium resistance was higher to selenate than to selenite, and strains that were more selenite-resistant were usually also more selenate-resistant. No differences in Se tolerance are apparent between isolates of *A. bisulcatus* or *S. pinnata*. Shoot endophytes from both plant species tended to be more sensitive to both selenite and selenate than root endophytes, sometimes even for isolates from the same genus (*Bacillus*). Comparison of the four most commonly isolated genera indicate that *Bacillus* is the most sensitive to selenite and selenate, while *Pantoea* is most resistant and *Pseudomonas* and *Staphylococcus* are intermediate in Se resistance.

**Table 2:**
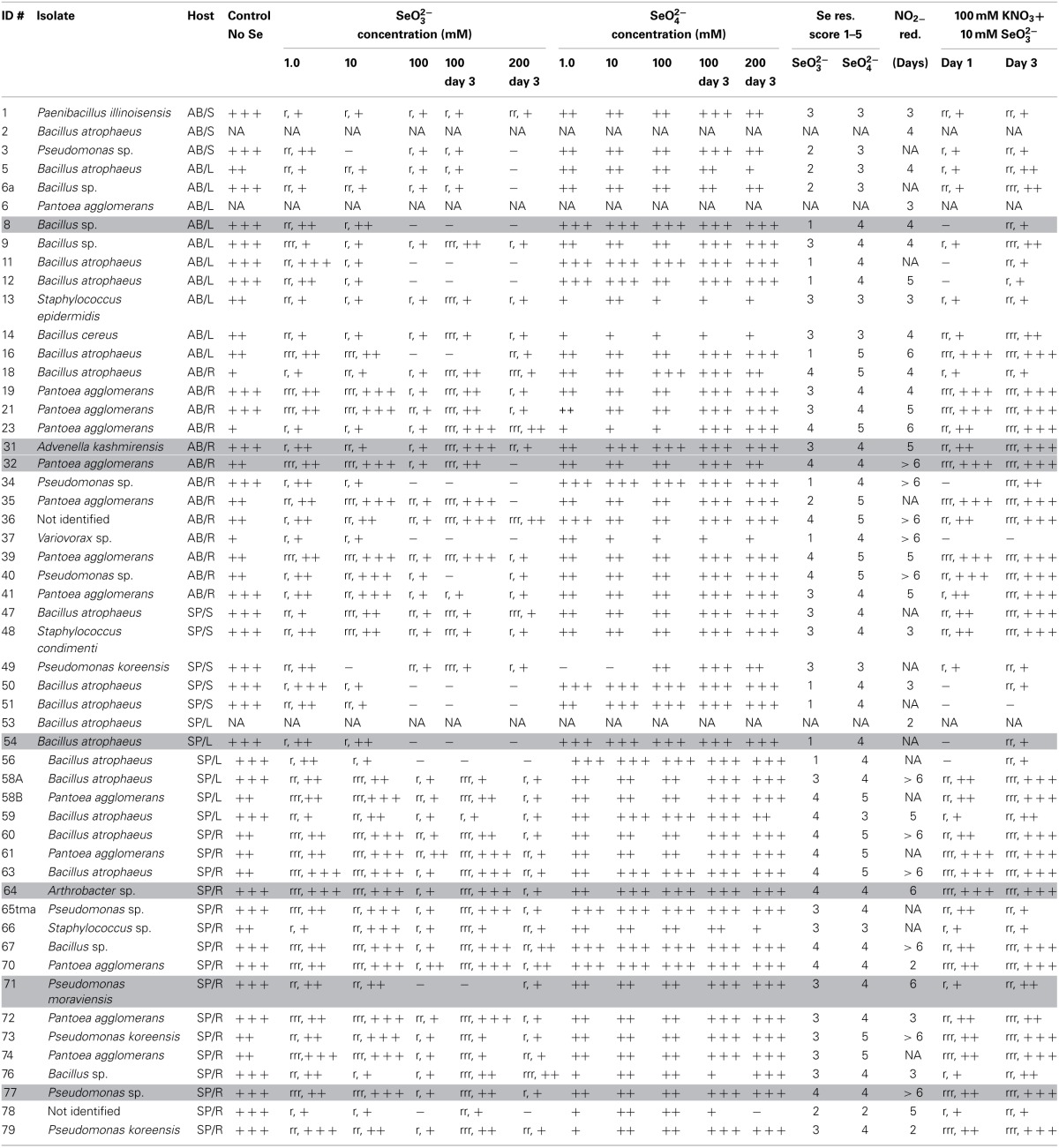
**Functional characterization of Se hyperaccumulator endophytes for abilities to grow on and/or reduce selenite and selenate, reduce selenite in the presence of nitrate, reduce nitrite and to produce siderophores**.

*Shown scores represent the average of three trials. The number of + signs (or −) denotes amount of growth after 1 day unless otherwise stated. The r, rr, and rrr indicate the amount of red coloration from elemental Se production. NA: No opportunity to observe. Growth on 0.1 mM Se are not shown for lack of space. Selenite and selenate resistance were scored on a scale from 1 to 5 as follows: 1, no growth above 10 mM Se. 2, no growth above 100 mM Se. 3, growth up to 200 mM Se with evidence of inhibition by Se. 4, growth up to 200 mM Se without inhibition. 5, growth up to 200 mM Se with apparent stimulation by Se. Nitrite reduction was scored as the number of days after which nitrite was reduced to below detectable level. If stated >6, nitrite reduction was measured but was not complete after 6 days. Gray rows indicate the isolates used for plant inoculation*.

Elemental Se production from 10 mM selenite was inhibited when 100 mM nitrate was added to the media for 20% of *A. bisulcatus* isolates and 60% of *S. pinnata* isolates. Growth was typically inhibited concomitantly. *Variovorax* sp. (*A. bisulcatus*, root) and *Bacillus atrophaeus* (*S. pinnata*, stem) were particularly sensitive to this inhibition (Table 2).

All tested isolates were able to reduce nitrite. Some of the isolates had completely reduced the supplied nitrite in 2 days, while some needed more than 6 days (Table 2). No apparent differences were found between isolates of *A. bisulcatus* or *S. pinnata* in this respect. The shoot endophytes from both species tended to reduce nitrite faster than the root endophytes. Among isolates reducing all nitrite in 2 days were *Bacillus atrophaeus* (*S. pinnata*, leaf), *Pantoea agglomerans* (*S. pinnata*, root) and *Pseudomonas koreensis* (*S. pinnata*, root). The endophytic isolates were also screened for their ability to produce siderophores. All isolates tested, from both *A. bisulcatus* and *S. pinnata* were able to produce siderophores.

### Inoculation of non-hyperacumulating plants by selected endophytes

A selection of seven bacterial endophytes was tested for their effects on plant growth and Se accumulation from naturally seleniferous soil: *Bacillus sp*. (*A. bisulcatus*, leaf), *Advenella kashmirensis* (*A. bisulcatus*, root), *Pantoea agglomerans* (*A. bisulcatus*, root), *Bacillus atrophaeus* (*S. pinnata*, leaf), *Arthrobacter* sp. (*S. pinnata*, root), *Pseudomonas moraviensis* (*S. pinnata*, root), and *Pseudomonas* sp. (*S. pinnata*, root). These isolates were chosen based on their capacity to grow on both selenate and selenite (10 mM), and to represent the microbial diversity and diversity in origin from roots and shoots of both HA species. For more information about the properties of the selected isolates, see Table 2. The endophytes originating from *A. bisulcatus* were inoculated to plants of crop species *Medicago sativa* from the same family (Fabaceae), while endophytes originating from *S. pinnata* were inoculated to plants of related crop species *Brassica juncea*. These crop species were chosen because they were not likely to already harbor these endophytes and because of the possible economic importance related to endophyte-enhanced Se uptake and growth of these plant species for biofortification or phytoremediation.

**Figure 6:**
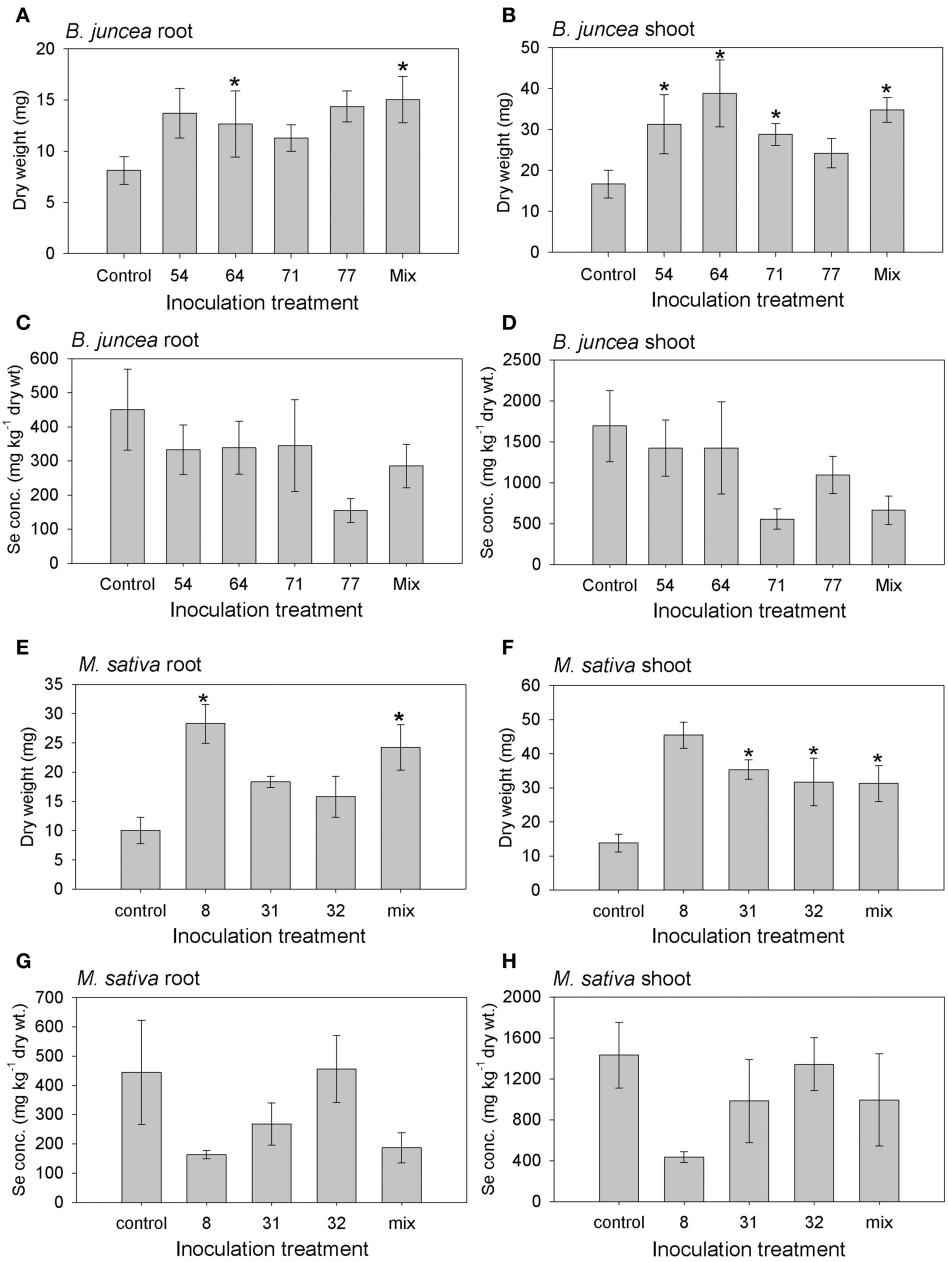
**Results from inoculation of *M. sativa* and *B. juncea* with endophytic bacteria isolated from Se hyperaccumulators *A. bisulcatus* (#8, 31, 32) and *S. pinnata* (#54, 64, 71, 77), respectively**. Additional treatments consisted of a mix of all 3 or 4 isolates, and an uninoculated control. **(A)** *B. juncea* root dry weight; **(B)** *B. juncea* shoot dry weight; **(C)** *B. juncea* root Se concentration; **(D)** *B. juncea* shoot Se concentration; **(E)** *M. sativa* root dry weight; **(F)** *M. sativa* shoot dry weight; **(G)** *M. sativa* root Se concentration; **(H)** *M. sativa* shoot Se concentration. Shown values represent mean and standard error of the mean (*n* = 6). Asterisks denote significant differences as compared to the control treatment (*p* < 0.05, Dunnett's multiple comparison test).

The inoculated plants of both plant species generally showed an increase in root and shoot biomass production, relative to the uninoculated controls; the effect on shoot biomass was significant for all but one inoculation treatment, while root biomass was significantly affected by two of the inoculation treatments for both species (Figures 6A,B,E,F). The positive effect of inoculation on plant growth was up to three-fold for individual isolates; there was no synergistic effect of inoculation with a mix of isolates. There were no significant differences in root or shoot Se concentration between control and inoculated plants of both plant species, although the inoculated plants on average showed lower Se levels (Figures 6C,D,G,H).

To get better insight into the mechanisms underlying the observed increase in plant biomass in the presence of these endophytic bacteria, the isolates were tested for various known plant growth promoting properties. Six out of the seven isolates tested (all except *Arthrobacter* sp.) showed phosphate solubilization activity, which may help plant hosts acquire this macronutrient (Qureshi et al., 2012). Five of the isolates produced acetoin (Table 3). This compound, produced by many bacteria during colony formation, has been shown to promote plant growth (Ryu et al., 2003). Furthermore, the two *Pseudomonas* spp. and *Pantoea agglomerans* produced the plant growth regulator indole acetic acid (IAA), which induces root formation and cell expansion (Bonner and Bandurski, 1952). Protease activity, a mechanism by which bacteria may help host plants defend against biotic stressors (Cho et al., 2007), was found for four of the isolates: both *Bacillus* species as well as *A. kashmirensis* and *Pseudomonas* sp. (Table 3). The two *Bacillus atrophaeus* isolates and to a lesser extent *Advenella kashmirensis* displayed chitinase activity (Table 3), which can protect plant hosts against insect herbivores (Thamthiankul et al., 2004) and fungal pathogens (Liu et al., 2002). None of the strains displayed acid production in a methyl red assay (Glick, 1995).

**Table 3:** **Plant growth promoting properties of the selected endophytes used for plant inoculation studies**.

**vv**	**Plant source**	**ID**	**Chitinase activity**	**Phosphate solubilization**	**Protease activity**	**Acetoin**	**Indole acetic acid**
#8	AB/L	*Bacillus* sp.	+++	+−	+++	+++	−
#31	AB/R	*Advenella kashmirensis*	−	+++	+++	+++	−
#32	AB/R	*Pantoea agglomerans*	−	+++	−	+++	+++
#54	SP/L	*Bacillus atrophaeus*	+++	+−	++	+++	−
#64	SP/R	*Arthrobacter* sp.	−	−	−	−	−
#71	SP/R	*Pseudomonas moraviensis*	−	+++	−	−	+++
#77	SP/R	*Pseudomonas* sp.	−	+++	+++	+++	+++

*For other properties of these accession numbers see Table 2. AB, isolated from Astragalus bisulcatus; SP, isolated from Stanleya pinnata; L, leaf; R, root*.

## Discussion

Plant-microbe interactions of hyperaccumulator plant species is still a relatively unexplored area (Alford et al., 2010). The objectives of this study were to determine how endophytic microbial composition compares between Se hyperaccumulator (HA) species and non-hyperaccumulator species, and to characterize endophytic bacteria from Se hyperaccumulators. The main findings from T-RFLP analysis were that bacterial endophyte diversity represented by T-RFs pattern was comparable between Se HA and related non-HA growing on the same seleniferous site. T-RFs composition appeared most similar between individuals of the same species. Beyond the species level, there was no apparent correlation of T-RFs composition with taxonomy, nor with plant Se concentration or spatial proximity in the field. The main findings from the HA endophyte characterization were that both species harbored a variety of culturable endophytes, comprising 66 morphotypes belonging to eight genera. The two plant species yielded similar numbers of endophytes, and these showed similar overall patterns. More endophytes could be cultivated from roots than shoots. The endophytes were highly Se resistant (up to 200 mM generally), especially the root endophytes. All could reduce selenite to elemental Se. Selected endophytes showed evidence of plant growth promoting properties, both in *in vitro* assays as well as *in vivo* in plant growth studies.

The finding that there was no apparent relation between endophyte colonization and plant Se status (judged from T-RFLP analysis) suggests there is no cost of Se hyperaccumulation in terms of reduced endophyte colonization: Hyperaccumulators can enjoy the same benefits from bacterial endosymbionts as other plants. The finding that the T-RF pattern was most similar in individuals of the same species, even when located at a distance over 50 m indicates that each plant species hosts its specific consortium of endosymbionts that is transferred at least in part vertically, via the seed. Enclosed in seeds, endophytes may be dispersed in the field by animals or wind. In cases where endophytes reinoculate emerging seedlings from the rhizosphere, selective bacterial recruitment may be determined by root-released compounds, which may include selenocompounds (El-Mehdawi et al., 2012).

It is worth noting that T-RF pattern evaluation has several limitations in comparison to more informative next generation sequencing. Individual T-RF peaks may contain fragments with the same length but originating from different bacteria (*in silico* distinction is possible, e.g., by MICA3 software, Shyu et al., 2007). Also, since amplicons of plant plastid or mitochondrial DNA may be present after PCR, these may affect the efficiency of bacterial DNA amplification. In our study, the RFUs of mitochondrial T-RFs were at maximum 4.9× higher than other T-RFs in the samples. It cannot be excluded that the least abundant bacterial species were not amplified due to template competition in the PCR reaction. Despite these limitations, comparison of T-RF patterns can give a valuable indication of endophytic diversity and composition.

Among the genera found in the HA plants in this study (*Bacillus, Pantoea, Pseudomonas, Paenibacillus, Variovorax, Advenella, Arthrobacter*, and *Staphylococcus*) some have also been reported as endophytic genera by other authors. Durán et al. (2014) found *Bacillus, Paenibacillus, Klebsiella*, and *Acinetobacter* in Se-supplemented wheat plants. Weyens et al. (2009), Brader et al. (2014) and other authors Jackson et al. (2013), Pereira and Castro (2014), Truyens et al. (2014), Visioli et al. (2014) and Wang et al. (2014) have also identified several genera we isolated here as endophytic bacteria (*Bacillus, Pseudomonas, Arthrobacter, Variovorax, Rhizobium, Rhodococcus, Burkholderia, Sphingomonas, Enterobacter, Microbacterium, Agreia, Sthenotrophomonas, Kocuria, Agrobacterium, Pantoea*). The finding that Se HA plants growing in their natural seleniferous habitat harbor a variety of bacterial endophytes agrees with and extends the earlier reports that Se HA *Astragalus bisulcatus* contains *Rhizobiaceae* endophytes in root nodules as well as an endophytic fungus in seeds (Valdez Barillas et al., 2012). In the earlier studies these endophytes were hypothesized to be responsible for the high fraction (up to 30%) of elemental Se found in these plants in the field (Lindblom et al., 2013). Indeed, all endophytes isolated in the current study were able to produce elemental Se, at least from selenite. In the plant, endophytic microbes may encounter selenate (the main form present in soil), selenite (metabolic intermediate) as well as various forms of organic selenocompounds accumulated in Se HA species (methyl-selenocysteine, selenocystathionine, as reported by Freeman et al., 2006). Thus, it is possible that these bacterial endophytes contribute to the observed elemental Se within HA plants in the field. Incidentally, it is relevant to note that all the experiments conducted in this study were performed under aerobic conditions; oxygen levels in some plant tissues may be lower.

Most endophytes isolated from HA plants were resistant to at least 10 mM selenate and selenite and most could resist up to 200 mM selenate (Figure 5). It is difficult to estimate what concentration of Se they may encounter in the HA plants. These plants tend to accumulate Se in all plant parts including roots, stems, leaves and seeds, up to ~1.5% of DW (0.15% of FW, or 1500 mg Se per kg FW). As a reference, this corresponds to 20 mM selenite or selenate. The concentration in certain tissue types such as the epidermis may be higher (Freeman et al., 2006). Most Se in the plants is likely stored within the cells, while the endophytes may be present in between cells where Se levels are likely to be lower. It is also possible that some of these endophytes occur in the rhizosphere at times, e.g., if they are transferred horizontally. In the soil around these HA plants, the total Se concentration was measured in several earlier studies to be on average 15 mg kg^−1^, with a maximum of 100 mg kg^−1^ or ppm (Galeas et al., 2007; El-Mehdawi et al., 2011). Overall, the Se resistance displayed by the majority of these HA endophytes appears to be high enough to withstand the Se levels they are likely to encounter inside and around HA plants, with the possible exception of shoot *Bacillus* strains (Table 2, Figure 5). It is not clear why Se resistance was generally lower for shoot isolates than root isolates, considering that the Se levels are higher in the shoots than the roots of HA. Perhaps the root endophytes occur in locations where they encounter more plant Se (e.g., in the xylem, a transport route for Se from root to shoot) while in leaves the Se is sequestered in more discrete, symplastic locations (vacuoles, trichomes) away from endophytes. More studies are needed to assess whether the high degree of Se resistance observed for these HA endophytes is uncommon. For comparison, in a study by Di Gregorio et al. (2006) where rhizosphere bacteria were subjected to a period of selection for resistance to selenate and selenite, the reported resistance achieved was similar to that found for the Se HA endophytes here, and was also higher for selenate than selenite. Selenate/selenite resistance abilities of endophytic bacteria have also been described by Durán et al. (2014), who showed that strains isolated from Se-supplemented wheat were tolerant to Se levels ranging from 60 to 180 mM. Thus, the level of Se resistance observed in our current study, while high, may not be extraordinary. Nevertheless, based on our preliminary (unpublished) data, endophytes from non-HA plants on seleniferous soil and from non-HA from non-seleniferous soil appear less Se resistant than the endophytes reported here from HA plants. A more thorough comparison is in progress, which should offer some interesting insight into whether HA plants may select for endophyte Se resistance.

The Se resistance of the isolates was higher for selenate than for selenite, which may be due to different levels of bacterial uptake or extrusion rates, or different detoxification mechanisms. It is interesting to note that strains that were more selenite-resistant were generally also more selenate-resistant, which may indicate that some of the bacterial resistance mechanisms are shared for both Se oxyanions. Our finding that these HA endophytes can all convert selenite into elemental Se, while none can do the same for selenate is not unexpected. Many bacteria can reduce selenite, while selenate reduction is rare (Vallini et al., 2005; Hunter and Manter, 2009; Mishra et al., 2011; Staicu et al., under revision). The conversion of selenite to elemental Se may serve as a bacterial detoxification mechanism (Kessi et al., 1999), since endophyte growth was never stimulated by selenite in our studies. The mechanism of selenite reduction to elemental Se in these endophytes remains to be determined. Bacteria are known to be able to use different selenite reduction mechanisms, which may involve hydrogenase (Yanke et al., 1995), arsenate reductase (Afkar et al., 2003), nitrate reductase (Avazeri et al., 1997), nitrite reductase (Bledsoe et al., 1999), glutathione reductase or thioredoxin reductase (Hunter, 2014). In addition, Zawadzka et al. (2006) showed that siderophores of *Pseudomonas stutzeri* KC were able to detoxify Se and tellurium oxyanions in bacterial cultures. All cultivable isolates in our study produced siderophores, which in some cases may have contributed to their Se resistance. All our endophyte isolates were also able to reduce nitrite, so nitrite reductase may also be a mechanism some of them employ for selenite reduction. Nitrate inhibited selenite reduction and, hence, growth for some of our isolates but not others (Table 2), which may indicate the selenite reduction mechanisms are not the same for all isolates. The capacity of bacteria to reduce selenite may be applicable for treatment of wastewater as well as for the production of Se nanoparticles for industrial purposes (Staicu et al., under revision). Polluted water from oil refineries can contain 20–30 mg Se L^−1^ in the form of selenite (Hansen et al., 1998), and bacteria such as those described in this study may be applicable for the treatment of such wastewaters.

Besides potentially being useful by themselves, endophytic bacteria may also be useful via their positive effects on plant growth, nutritional quality and elemental accumulation. Plant-associated bacteria can affect the efficiency and rate of phytoextraction of trace elements in contaminated soils (Sessitsch et al., 2013). In the case of Se this is relevant for phytoremediation and biofortification, since Se can both be toxic and serve as a nutrient. The seven isolates that were tested in this study showed a general tendency to enhance the growth of *M. sativa* and *B. juncea*, while not significantly affecting Se accumulation (Figure 6). The possible mechanisms by which these endophytes caused the observed positive effect on plant growth may lie in the observed chitinase activity, phosphate solubilization activity, protease activity, acetoin production and/or IAA plant growth hormone production (Table 3). The different isolates differed with respect to these activities. Another potential plant growth promoting property that all isolates displayed was the production of siderophores, possibly aiding plants in acquiring nutritional iron from soil. This may explain why one isolate (#64, Table 3) did not display any of the other plant growth promoting properties, yet still had a positive effect on the plant growth. It remains to be determined whether this plant growth stimulation by these endophytes is Se-dependent or not, but based on preliminary (unpublished) results from plants grown on gravel and supplied with or without selenate, the positive effect on growth occurs both with and without selenate.

While the shoot and root Se levels of *M. sativa* and *B. juncea* were not significantly different between inoculated and control plants, it is worth noting that on average the inoculated plants contained lower tissue Se levels in this experiment (Figure 6). More experiments are needed to determine whether this trend may be significant with more replication. It is feasible that bacterial endophytes volatilize Se from the plant tissues, thus causing lower Se levels in inoculated plants. It is also possible that the inoculated bacteria were also present in the rhizosphere, and there reduced plant Se bioavailability by, e.g., elemental Se production, or competed with the plant for Se uptake. Some of the Se in this seleniferous soil, which was collected from around Se hyperaccumulators in the field, may have been in organic forms (El Mehdawi et al., 2015). This organic Se may constitute an attractive carbon source for rhizosphere bacteria. If indeed a substantial fraction of the Se in this soil was organic, this may also explain the high plant tissue Se levels observed (Figure 6). *Brassica juncea* has been shown in earlier studies to reach much higher bioaccumulation levels from organic forms of Se than from selenate or selenite (Zayed et al., 1998; de Souza et al., 2001). Preliminary results from plants grown on gravel and supplied with selenate indicate that inoculation with these same endophyte strains can cause plant Se levels to be enhanced. More extensive studies are needed to confirm this, but it is feasible that endophyte inoculation affects plant Se accumulation differently, depending on the form of Se supplied. In an earlier study by de Souza et al. (1999) rhizosphere bacteria from a seleniferous area were shown to enhance Indian mustard growth as well as selenate uptake and volatilization. The mechanism likely involved stimulated root hair production, perhaps via bacterial IAA production. Furthermore, Di Gregorio et al. (2006) showed that certain bacteria from the plant rhizosphere or endosphere can have positive effects on plant Se decontamination through either phytoextraction or putative volatilization on Se-rich soil. More recently, several soil bacteria isolated from a polluted area were shown to enhance wheat growth and Se accumulation (Yasin et al., 2015). More studies are needed to determine the long-term effects of these endophyte strains on growth and Se accumulation of different plant species, growth substrates and forms of Se supplied. Such studies will be useful to determine the potential of these strains to enhance the efficiency of Se biofortification and phytoremediation practices. If indeed these strains can enhance plant growth and/or affect Se accumulation, this will be particularly useful for phytoextraction, since this type of phytoremediation usually requires several years to clean up the contaminated site (Macek et al., 2000; Pilon-Smits, 2005; Mackova et al., 2009; Vangronsveld et al., 2009). Future studies with different plant-microbe combinations may also help shed more light on the individual interactions between plants and individual or combinations of microbes. This may allow us to optimally employ the symbiotic synergisms between plants and their microbiomes for phytoremediation.

### Conflict of interest statement

The authors declare that the research was conducted in the absence of any commercial or financial relationships that could be construed as a potential conflict of interest.

## Supplementary material

Supplemental Table 1 **Matrix scoring the presence or absence of each T-RF in each individual plant**. The presence of T-RF is represented by “1,” the absence of the T-RF is represented by “0.” Column A represents the size of T-RFs. Ab4, Ab5, Ab10–samples of hyperaccumulators *Astragalus bisulcatus*; Sp5, Sp14, and Sp30–samples of hyperaccumulators *Stanleya pinnata*; Ms25, Ms26–samples of non-hyperaccumalating plants of *Medicago sativa* L.; Pb22, Pb23, Pb24–samples of non-hyperaccumalating plants of *Physaria bellii* G. Mulligan.

Supplemental Table 2 **Macroscopic and microscopic characterization of individual endophytic isolates used for detrended canonical correspondence analysis (CANOCO)**. The presence of studied characteristic is represented by “1,” the absence is represented by “0.” Row 1 describes characteristic, row 2 represent the abbreviation used in the analysis.
